# Hydrogels for Climate Change Mitigation: Applications in Water Harvesting, Passive Cooling, and Environmental Solutions

**DOI:** 10.3390/gels11110924

**Published:** 2025-11-19

**Authors:** Julia Gałęziewska, Weronika Kruczkowska, Katarzyna Helena Grabowska, Żaneta Kałuzińska-Kołat, Elżbieta Płuciennik

**Affiliations:** 1 Department of Functional Genomics, Faculty of Medicine, Medical University of Lodz, Żeligowskiego 7/9, 90-752 Lodz, Poland; julia.galeziewska@stud.umed.lodz.pl (J.G.); weronika.kruczkowska@stud.umed.lodz.pl (W.K.); katarzyna.grabowska1@stud.umed.lodz.pl (K.H.G.); zaneta.kaluzinska@umed.lodz.pl (Ż.K.-K.); 2 Department of Biomedicine and Experimental Surgery, Medical University of Lodz, Narutowicza 60, 90-136 Lodz, Poland

**Keywords:** hydrogels, climate change mitigation, water harvesting, passive cooling, environmental application

## Abstract

Climate change presents significant global challenges, with rising temperatures, extreme weather events, and degrading ecosystems threatening both human societies and the environment. The increasing intensity of these climatic effects demands innovative approaches to adaptation and mitigation. Hydrogels, three-dimensional networks of crosslinked polymers with water absorption and retention properties, have become viable multipurpose materials for climate solutions in response to these pressing issues. This review examines four primary applications of hydrogels as climate technologies: atmospheric water harvesting, passive cooling, soil health enhancement, and energy conservation. These materials address climate challenges through their unique properties including high water absorption capacity, stimuli-responsive behavior, and biocompatibility. By effectively capturing moisture, hydrogel-based devices provide sustainable freshwater production in areas with limited water resources. For thermal management, they offer passive cooling through evaporative processes, reducing energy consumption compared to conventional air conditioning systems. Superabsorbent hydrogels in agriculture help drought-resistant crop development in arid areas and improve soil water retention. Smart windows with thermochromic hydrogels allow for passive energy savings by dynamically modulating the sun’s light without the need for additional electricity. Through integrated deployment techniques, biodegradable formulations from sustainable sources handle various climate issues while ensuring environmental compatibility.

## 1. Introduction

Climate change represents one of the most pressing global challenges of our time, with rising temperatures, increasing extreme weather events, and deteriorating ecosystems threatening both human societies and natural environments [1]. The Earth’s average temperature has increased by approximately 1 °C since the late 1800s, with 2024 recording temperatures 1.55 °C ± 0.13 °C warmer than pre-industrial levels (1850–1900), making it the warmest year in the 175-year observational record [2,3,4]. Sea levels have risen by about 24 cm and are projected to continue rising. Atmospheric concentrations of key greenhouse gases, including carbon dioxide (CO_2_), methane (CH_4_), and nitrous oxide (N_2_O), reached their highest levels in at least 800,000 years in 2023, with CO_2_ accounting for approximately 66% of the heat absorbed by long-lived greenhouse gases [5,6].

Traditional approaches to climate challenges face significant limitations. Water scarcity management relies on energy-intensive reverse osmosis desalination (3–4 kWh/m^3^) and refrigeration-based atmospheric water generation, both unsuitable for off-grid applications [6,7,8,9]. Conventional air conditioning systems account for 10% of global electricity consumption and are projected to triple by 2050, creating a paradox where cooling needs increased emissions. In agriculture, traditional irrigation systems consume 70% of global freshwater with substantial losses through evaporation, while chemical amendments contribute to environmental degradation [10,11,12]. Building energy conservation typically relies on static smart windows requiring external power and complex control systems [13].

In the face of these challenges, innovative solutions are urgently needed to mitigate and adapt to the effects of climate change (Table 1). Hydrogels-three-dimensional (3D) networks of crosslinked polymers with water absorption and retention capabilities-have emerged as promising materials for addressing multiple facets of the climate crisis. Their unique properties, including high water absorption capacity, stimuli-responsive behavior, and biocompatibility, make them particularly valuable for climate solutions [7,8,14,15].

Hydrogels offer distinct advantages over conventional approaches: they can absorb hundreds to thousands of times their dry weight in water through passive mechanisms, eliminating energy penalties [15], provide evaporative cooling with up to 90% water content without electrical input [16,17], reduce agricultural irrigation requirements by 30–50% while improving yields [18,19], and enable autonomous building energy conservation reducing consumption by up to 45% [20]. Importantly, hydrogels provide multifunctional integration, where a single system addresses water harvesting, cooling, soil improvement, and energy conservation simultaneously [21,22]. Their biodegradable nature from renewable sources ensures environmental compatibility and supports circular economy principles [23]. Hydrogels demonstrate superior energy efficiency metrics compared to conventional technologies: atmospheric water harvesting via hydrogels requires 0.1–0.5 kWh/m^3^ versus 3–4 kWh/m^3^ for reverse osmosis; passive cooling through hydrogel evaporation achieves temperature reductions of 5–15 °C with zero energy input compared to air conditioning systems that consume 2–5 kW for similar cooling loads. However, critical limitations include lower absolute water production rates (2–5 L/m^2^/day for hydrogels vs. 100–1000 L/m^2^/day for desalination) and dependence on ambient humidity levels above 30% for effective operation [24,25].

This review explores the potential of hydrogels as materials for climate change mitigation and adaptation, focusing on their applications in water harvesting, passive cooling, soil health improvement, and energy conservation. We describe different types of hydrogels, their features, and how they can be leveraged to address climate-related challenges. Furthermore, we discuss eco-friendly innovations in hydrogel technology and synergistic approaches that combine hydrogels with other sustainable solutions to maximize their impact on climate change mitigation and adaptation strategies. The following sections will demonstrate how hydrogels represent not just an alternative to conventional approaches, but a technology that can redefine our response to the climate crisis through integrated, sustainable, and effective solutions.

**Table 1:** Benchmark Table—reported performance metrics and standard test protocols for hydrogel-based climate applications.

Application	Representative System	Performance Metrics	Standard Test Conditions	Cycle Protocol	References
**Atmospheric water harvesting (AWH)**	PNIPAm- or alginate-based sorbent hydrogels; MOF-hydrogel hybrids; salt-polymer composites	Typical water yield: 2–5 L m^−2^ day^−1^; Max reported: 14.9 L m^−2^ day^−1^ (60–80% RH, 20–35 °C)	RH 30–90%; ambient 25–40 °C; desorption 40–70 °C (solar)	≈3 h adsorption + ≈3 h desorption; up to 8 cycles day^−1^	[26,27,28,29]
**Passive cooling—evaporative**	Hydrogel evaporators/roof coatings	ΔT 5–15 °C (surface-to-ambient); Cooling power 50–150 W m^−2^; Duration 3–8 h continuous (day), overnight rehydration	RH 40–80%; ambient 20–45 °C; daytime solar load; moderate wind	Continuous daytime; overnight water recharge; multi-day repeatable	[30,31,32,33,34]
**Soil water retention and yield**	Superabsorbent hydrogels in soil (e.g., PAM, natural-polymer blends)	Irrigation reduction 30–50%; Yield increase 11–51%; Absorption 100–1000 g g^−1^; Application 0.2–0.4 g per 100 g soil; Durability 100–500 hydration cycles	Field/greenhouse; crop-dependent; ambient 15–35 °C; irrigation per protocol	Per-crop growing season; rewet/dry cycles per irrigation schedule	[35,36,37,38,39,40,41,42]
**Energy conservation—smart windows**	Thermoresponsive (e.g., PNIPAm/HPC) hydrogel glazing	Solar modulation ΔT_solar_ up to ≈33%; Visible transmittance T_lum_ ≈ 95%; Energy reduction up to 45% (model/prototype)	LCST window 20–50 °C; indoor 20–26 °C; outdoor 10–35 °C; standard solar spectra	Reversible LCST cycling >100 cycles without degradation.	[43,44,45,46,47,48]
**CO_2_ capture**	Amine-functionalized hydrogel sorbents (e.g., PEI-hydrogel)	6.5 mol kg^−1^ (pure CO_2_); 0.7 mol kg^−1^ (air); 25 °C; 40% RH; ~30 min adsorption; ~60 °C desorption; ~4 h cycle	400 ppm CO_2_ (air tests) or pure CO_2_; ambient pressure; 25 °C; controlled RH (40%)	Batch adsorption/desorption; repeated cycling	[7,13]

(RH = relative humidity; LCST = lower critical solution temperature).

## 2. Hydrogel Type, Manufacturing and Features

### 2.1. Classification of Hydrogels

Hydrogels encompass a diverse range of materials classified based on their source, composition, preparation method, and cross-linking mechanisms. Understanding these classifications is essential for tailoring hydrogels to specific climate applications (Table 2).

#### 2.1.1. Hydrogels Classification by Polymer Source

Natural polymer hydrogels are derived from naturally occurring polymers such as polysaccharides and proteins, providing excellent biocompatibility and biodegradability. These materials have gained popularity due to their renewable origins and environmental friendliness [49,50]. Dextran, agarose, chitosan, alginate, collagen, and gelatin are examples of natural polymers that may be cross-linked to generate hydrogels [49,51].

Synthetic polymer hydrogels are manufactured from chemically synthesized polymers, offering greater control over properties and often superior mechanical strength compared to natural counterparts [10,11,49]. Polyacrylamide (PAM), polyethylene glycol (PEG), poly(vinyl alcohol) (PVA), and polyethylenimine (PEI) are some of the most common synthetic polymers utilized in hydrogel production [13,49,52]. In terms of climate solutions, synthetic hydrogels have shown incredible promise [7,53]. Polyelectrolyte hydrogels, for example, have been created for atmospheric water harvesting, with a water output of up to 2410 mL per kilogram of sorbent every day, making them a possible solution to freshwater scarcity in dry locations [15]. Polyethylenimine hydrogel particles can absorb 6.5 mol CO_2_ per kg in pure CO_2_ settings and 0.7 mol per kg in direct air capture [13].

Composite hydrogels represent an advanced approach, combining multiple polymers or incorporating additional components to create materials with enhanced properties [16,49,54]. Interpenetrating polymer networks (IPNs) are made up of two or more polymer networks that are partly connected but not covalently bound together [55]. Another example of such hydrogels is nanocomposite hydrogels that include nanoparticles into the polymer matrix, which significantly improve mechanical strength, electrical conductivity, and thermal characteristics [16,20,21,56].

**Table 2:** **Classification and Characteristics of Hydrogel Types for Climate Applications.** This table compares four major hydrogel categories based on polymer source and network architecture. Natural polymer hydrogels exhibit superior biocompatibility and biodegradability (2–12 months) but lower mechanical strength than synthetic variants. Synthetic hydrogels achieve higher water absorption capacities (100–1000× dry weight) with environmental persistence concerns. Composite/hybrid systems offer enhanced multifunctionality at increased synthesis complexity. Water absorption ranges from 10 to 100× dry weight for natural polymers to 500–2000× for advanced composites.

Hydrogel Type and Polymer Source	Climate Applications	Relative-Humidity (RH) Uptake	Regeneration	Outdoor Durability	Soil Cycling	Emissivity	References
**Natural** **Polymer:** **Chitosan, alginate, collagen, gelatin**	Soil amendment, water harvesting, passive cooling	0.1–0.3 g g^−1^ at <30% RH (limited)	60–80 °C, 180–250 kJ/mol (moderate efficiency)	2–12 months (enzymatic degradation, microbial attack)	6–18 months (full mineralization, enriches soil)	0.65–0.75 (transparent matrix, poor radiative cooling)	[16,35,54,57]
**Synthetic Polymer:** **Polyacrylamide, polyethylene glycol**	Atmospheric water generation, CO_2_ capture, energy storage	0.8–1.5 g g^−1^ at 30–50% RH (moderate)	40–60 °C, 200–350 kJ/mol (higher efficiency)	5–10 years (UV-resistant but persistent microplastics)	Non-applicable (non-biodegradable persistence)	0.45–0.60 (absorbed solar radiation, poor radiative window)	[53,54,58,59]
**Composite/Hybrid:** **Natural + synthetic combinations, nanoparticle incorporation**	Advanced cooling systems, smart windows, precision agriculture	2.8–3.5 g g^−1^ at 15–25% RH (excellent)	55–70 °C, 320–450 kJ/mol (highest efficiency)	6 months-2 years (salt crystallization stress + UV degradation)	Variable (depends on polymer component ratio)	0.70–0.85 (enhanced by salt crystalline structure)	[20,21,56,60,61]
**Interpenetrating Networks (IPNs):** **Two or more polymer networks**	Building materials, thermal regulation, water purification	0.5–1.2 g g^−1^ at 25–40% RH (moderate-good)	45–65 °C, 280–400 kJ/mol (balanced)	1–3 years (superior UV resistance vs. single polymers)	8–24 months (controlled mineralization)	0.55–0.75 (depends on composition)	[58,59]

#### 2.1.2. Hydrogels Classification by Manufacturing Approaches

**Figure 1:**
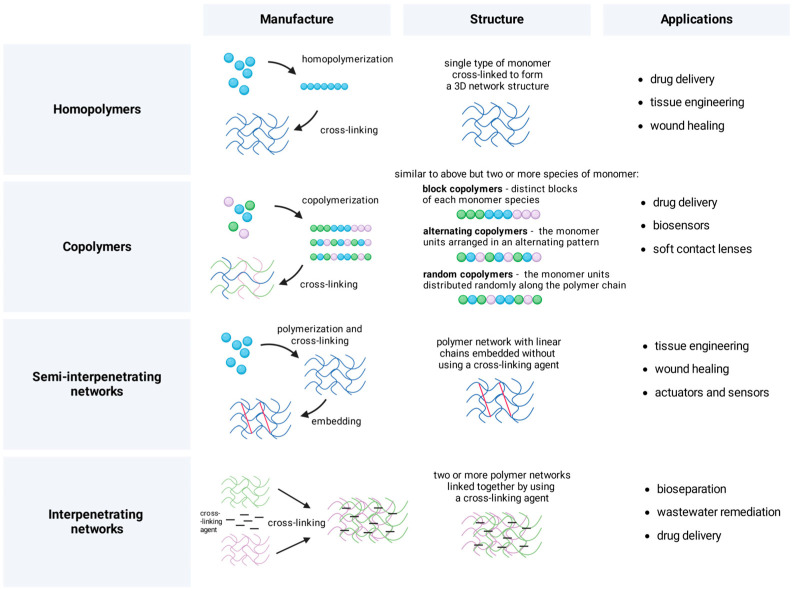
**Production, composition, and illustrative uses of polymeric materials.** The quantity of monomers in a formulation and their order determine its future use, such as in tissue engineering, medication delivery, and wastewater treatment. Figure created with BioRender.com (accessed on 12 April 2024). From *Kruczkowska* et al. *Biomedical Trends in Stimuli-Responsive Hydrogels with Emphasis on Chitosan-Based Formulations. Gels 2024, 10, 295. https:*//*doi.org*/*10.3390*/*gels10050295* [51].

Hydrogels may also be classed according to their cross-linking mechanisms, which are crucial to their network design and characteristics. Hydrogels fall into two categories: physically cross-linked and chemically cross-linked (Figure 1) [16,23]. Physically cross-linked hydrogels are formed by non-covalent interactions such as hydrogen bonding, ionic contacts, hydrophobic associations, or crystallization [16,55,62,63]. These physical cross-links produce reversible junctions that may reconstruct after breaking, frequently resulting in self-healing characteristics [55,64,65,66].

The primary methods for hydrogel fabrication include chemical and physical crosslinking approaches. Chemical crosslinking relies heavily on radical polymerization, where precise stoichiometric control governs the reaction [67,68]. Alternatively, radiation-based synthesis employs ionizing radiation or UV light to initiate cross-linking without chemical initiators—a scalable sheet production method where hydrophilic polymer mixes are coated onto liners and exposed to controlled radiation, eliminating contaminant risks while enabling active ingredient infusion [69,70]. Another chemical approach is grafting, which creates superabsorbent by functionalizing natural polymers like cellulose or starch with synthetic monomers like acrylonitrile and then hydrolyzing (saponifying) the resulting material [71]. Advanced fabrication processes include electrospinning for high-surface-area nanofibrous structures, 3D printing for complex geometries, and freeze-drying to create porous architectures [72,73,74]. These methods enable customizable porosity, degradation profiles, and functional loading to meet specific climate application requirements [75,76].

The choice of fabrication method directly impacts climate application performance (Table 3). Free radical polymerization produces heterogeneous pore distributions (10–100 nm) optimizing water harvesting mass transfer but creating irregular crosslinking that requires thermal energy for water release during regeneration [67]. Controlled polymerization (RAFT, ATRP) creates uniform nanopores (5–20 nm) that enhance water retention (>90% after 24 h evaporation) through increased capillary forces [30,31,68]. UV-initiated crosslinking forms photolabile bonds causing 40–60% property loss after 1000 h outdoor exposure (3–4 months), while thermal initiation avoids photodegradation [77]. Ionic crosslinking through multivalent cations enables regeneration at moderate temperatures (60 °C) and preserves performance through 500+ cycles, while 80 °C regeneration causes accelerated degradation after 200–300 cycles. Temperature-responsive hydrogels release up to 85% of absorbed water at approximately 60 °C through temperature-induced phase transitions [60,61]. Electrospinning produces exceptional surface area-to-volume ratios (up to 1000 m^2^/g) and aligned fiber structures enhancing water transport and evaporative cooling, with cooling powers of 50–150 W/m^2^ achieved under laboratory conditions [30,72,78,79,80,81]. Freeze-drying creates highly porous structures (80–95% porosity) with interconnected macropores enabling rapid water absorption and sustained soil water release [36,82]. Radiation-induced crosslinking eliminates chemical initiator residues, addressing food safety concerns for agricultural applications [69,83]. Gradient crosslinking with spatially varying network densities provides enhanced mechanical robustness, while self-healing mechanisms incorporating reversible bonds enable autonomous repair of microcracks, with optimization strategies extending operational cycles by 2–3× while maintaining 90% performance retention [66,77]. This synthesis-performance relationship enables rational material selection: controlled polymerization for enhanced water retention in passive cooling, electrospinning for maximum cooling power through surface area, freeze-drying for agricultural water retention with rapid absorption, radiation crosslinking for food-safe soil amendments, and gradient/self-healing approaches for extended durability in cycling applications.

#### 2.1.3. Advanced Synthesis and Comparative Analysis of Fabrication Methods

Recent advances in smart hydrogel systems have emphasized multi-responsive materials that integrate multiple stimuli-sensing mechanisms within a single platform. These intelligent systems combine temperature, pH, light, and magnetic responsiveness to enable sophisticated climate control applications. For instance, dual temperature-pH responsive hydrogels can autonomously regulate both thermal and chemical conditions in agricultural applications. Photothermal-responsive hydrogels incorporating photothermal agents enable on-demand actuation with switching times reduced from hours to seconds. The molecular mechanisms underlying temperature responsiveness involve precise balance between hydrophilic and hydrophobic interactions: below the Lower Critical Solution Temperature, polymer-water hydrogen bonds dominate, while above the LCST, hydrophobic interactions drive chain collapse. This understanding enables rational design with precisely tuned transition temperatures. Shape-memory hydrogels represent another emerging class, capable of fixing temporary shapes and recovering upon stimulation, offering opportunities for adaptive architecture in climate-responsive buildings [88].

Electrospinning produces hydrogels with exceptional surface area-to-volume ratios (up to 1000 m^2^/g) and aligned fiber structures that enhance mechanical anisotropy, making them ideal for applications requiring directional properties [72,78,79,80,81]. 3D printing offers unprecedented design flexibility and the ability to create complex geometries with spatial control over composition and properties, but current printing resolutions are limited to 10–100 μm, restricting fine-scale structural control. Freeze-drying techniques create highly porous structures (porosity: 80–95%) with excellent water absorption [82].

### 2.2. Unique Features and Property Characterization of Hydrogels

Hydrogels have a mix of properties that make them extremely flexible materials for a variety of applications, notably in tackling climate-related concerns.

#### 2.2.1. Biocompatibility and Biodegradability

One of the most notable advantages of hydrogels, especially those made from natural polymers, is their high biocompatibility and biodegradability [49,55]. Hydrogels’ hydrophilic nature generates an environment analogous to biological tissues, making them ideal for use with living systems [89]. The biodegradability of hydrogels, particularly those produced from natural sources, addresses rising environmental concerns about plastic pollution and material durability. In agricultural applications, biodegradable hydrogels can increase soil quality and crop development while leaving no permanent synthetic residues that could affect soil ecosystems [90].

#### 2.2.2. Stimuli-Responsive Behavior

An important feature of many advanced hydrogels is their ability to respond to environmental stimuli such as pH, temperature, light, or specific chemical triggers [59]. This stimuli-responsive behavior enables smart systems that can adapt to changing conditions without requiring external control mechanisms [91].

Temperature-responsive hydrogels undergo volume phase transitions at specific temperatures, either contracting or expanding based on their design [92,93]. This property is particularly valuable for atmospheric water harvesting systems, where temperature fluctuations between day and night can drive water absorption and release cycles [60,61,94].

#### 2.2.3. Swelling Behavior and Kinetics

Swelling behavior is fundamental to hydrogel performance, governed by the balance between osmotic pressure driving water uptake and elastic restoring forces of the polymer network [95,96].

The equilibrium swelling ratio (ESR) is determined by the Flory-Rehner equation, which accounts for polymer-solvent interactions (χ parameter), cross-linking density (ν_e_), and polymer volume fraction in the swollen state. Temperature-sensitive hydrogels exhibit volume phase transitions at critical temperatures, with swelling ratios changing by orders of magnitude within narrow temperature ranges (typically 2–5 °C) [97].

#### 2.2.4. Water Retention and Release Mechanisms

Water retention in hydrogels involves multiple mechanisms including bound water (hydrogen bonding with polymer chains), semi-bound water (influenced by polymer network structure), and free water (bulk water within pores) [57,98,99]. Gunko et al. comprehensively analyzed water states in hydrogels, classifying water into strongly bound (SBW, unfrozen at T < 260–265 K), weakly bound (WBW, unfrozen at 260–265 K < T < 273 K), and non-bound with nearly bulk water properties (NBW, frozen at 273 K) [57].

Controlled water release is achieved through various mechanisms including diffusion-controlled release, swelling-controlled release, and erosion-controlled release [100]. Liu et al. reviewed hydrogels for long-term moisture retention under ambient conditions, elucidating multiscale water-evaporation inhibition mechanisms through thermodynamics governing free and bound water states [101].

#### 2.2.5. Thermal Conductivity and Thermal Stability

Thermal conductivity of hydrogels typically ranges from 0.4 to 0.6 W/m·K, significantly higher than dry polymers (0.1–0.2 W/m·K) due to the presence of water.

Thermal stability varies significantly depending on the polymer backbone and cross-linking chemistry. Natural polymer hydrogels typically decompose at 200–300 °C, while synthetic hydrogels can withstand temperatures up to 400–500 °C before significant degradation occurs [102,103].

#### 2.2.6. Mechanical Properties and Stability

Mechanical properties of hydrogels are strongly dependent on cross-linking density, polymer concentration, and water content. Typical elastic moduli range from 0.1 kPa to 10 MPa, with compressive strengths varying from 10 kPa to 10 MPa. Double-network hydrogels achieve exceptional mechanical properties, with tensile strengths exceeding 1 MPa and fracture energies comparable to cartilage. Mechanical stability under cyclic loading is crucial for practical applications. Hydrogels exhibit viscoelastic behavior with time-dependent stress relaxation and creep. The mechanical fatigue resistance can be enhanced through introducing sacrificial bonds that break preferentially under stress, preserving the primary network structure [104].

#### 2.2.7. Photothermal Properties and Solar Responsiveness

Photothermal hydrogels incorporate light-absorbing components such as carbon nanotubes, graphene oxide, or metallic nanoparticles to convert light energy into heat. These materials exhibit photothermal conversion exceeding 80% efficiency and can achieve rapid temperature increases (>20 °C) under solar irradiation. Solar-responsive hydrogels demonstrate autonomous actuation under natural sunlight, enabling self-regulating systems for water management and environmental control. The integration of photosensitive molecules allows for precise control over swelling behavior through light wavelength and intensity modulation. These systems show particular promise for water evaporation applications, with some achieving evaporation rates exceeding 2 kg·m^−2^·h^−1^ under solar irradiation [105,106,107].

### 2.3. Sustainability and Current Challenges in Hydrogel Technology

Despite significant advances in hydrogel technology, several critical challenges limit their widespread practical implementation.

A fundamental challenge in hydrogel design is the inherent trade-off between water absorption capacity and mechanical strength. High water content typically leads to poor mechanical properties, while strong mechanical networks often exhibit limited swelling capacity [108]. Current approaches to address this limitation include double-network designs and nanocomposite reinforcement, but these solutions significantly increase complexity and cost [11]. Long-term stability remains a major concern, particularly for applications requiring extended operational lifetimes. Hydrogels are susceptible to various degradation mechanisms including oxidation, UV radiation, freeze–thaw cycles, and microbial attack [109]. Natural polymer hydrogels are particularly vulnerable to enzymatic degradation and microbial colonization, which can lead to rapid performance deterioration [110].

Response time and kinetics represent another significant limitation, especially for stimuli-responsive hydrogels. Many applications require rapid response to environmental changes, but current hydrogel systems often exhibit response times of minutes to hours rather than seconds [111,112].

The translation from laboratory-scale synthesis to industrial production presents numerous challenges. Many advanced hydrogel formulations require precise control over reaction conditions, specialized equipment, and expensive raw materials that are difficult to scale economically [113]. Batch-to-batch variability in properties remains a significant issue, particularly for complex composite systems [114]. Health and safety concerns associated with hydrogel components, particularly cross-linking agents and additives, need thorough evaluation. Several synthetic cross-linking agents are commonly used but are characterized as being harmful and toxic to both humans and the environment. Some crosslinking agents produce large amounts of toxic by-products that require elimination by extensive washing, thereby affecting hydrogel biocompatibility and environmental safety [115,116].

The mechanisms underlying stimuli-responsive behavior, particularly for multi-responsive systems, require deeper investigation [117]. Future research efforts should focus on addressing critical limitations in current hydrogel systems through targeted investigations and technological developments. Development of next-generation mechanical reinforcement strategies is urgently needed, with specific targets including achieving tensile strengths >10 MPa while maintaining swelling ratios >500%, enabling applications in demanding mechanical environments [118].

Rapid-response hydrogel systems with response times <1 min are essential for real-time environmental applications [112]. This requires fundamental research into transport mechanisms, network design, and triggering strategies that can accelerate stimulus-response kinetics [119]. Machine learning approaches show particular promise for optimizing complex multi-component systems, with AI revolutionizing hydrogel design by expediting material discovery and enabling precise customization. Recent advances demonstrate specificity of 88.4% and sensitivity of 86.2% in predicting hydrogel properties [120].

## 3. Climate Solutions Powered by Hydrogels

Climate change is one of the most significant global concerns of our day, and it is brought on by human activity such as the burning of fossil fuels, deforestation, and industrial operations. Since the late 1800s, the Earth’s average temperature has increased by nearly 1 °C. The highest ocean heat content in the 65-year observational record was also recorded in 2024, 16 ± 8 ZJ greater than the previous mark, which was established in 2023. Sea levels have risen by about 24 cm and are expected to rise more [121,122].

**Figure 2:**
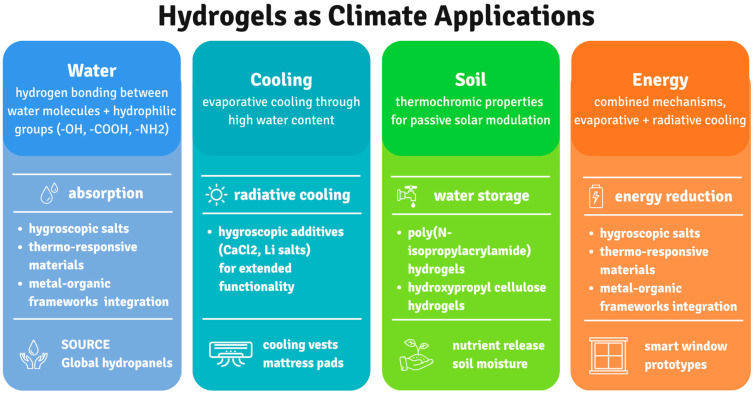
**Hydrogels as Climate Applications.** Hydrogels as multifunctional climate mitigation materials across four key application domains. There are several climate-beneficial properties of hydrogels. For water applications, they absorb large amounts of water via hydrogen bonding, as seen in SOURCE Global’s solar hydropanels. In cooling systems, hydrogels provide evaporative and radiative cooling, enhanced by hygroscopic additives like CaCl_2_ for personal cooling and building thermal management. For soil, they offer passive solar modulation through thermochromic properties and improve nutrient release, heavy metal remediation, and moisture retention with advanced formulations like PNIPAm and HPC hydrogels. Energy applications combine these cooling and absorption features with hygroscopic salts and thermo-responsive materials to address water scarcity and agriculture. Overall, hydrogels, with their integrated hygroscopic salts, thermo-responsive materials, and metal–organic frameworks, are versatile platforms for tackling various climate challenges through optimized molecular design. *Figure created using Canva ver. 2025 (accessed on 5 November 2025)*.

Climate change challenges require innovative approaches, and hydrogels present a possible path (Figure 2). These special materials, made of networks of water-rich polymers, are being created and used for important climate-related applications like environmental remediation, carbon emissions absorption, and renewable energy production enhancement. Hydrogels are useful weapons in the fight against climate change effects because of their inherent versatility [123,124,125].

### 3.1. Hydrogels in Water Harvesting

Water scarcity affects billions of people worldwide, with more than 2 billion people lacking access to safe water according to the United Nations. The countries with the worst water access issues in healthcare institutions include Tajikistan and Liberia (both at 49%), Ethiopia (45%), Kyrgyzstan (57%), and Guinea (68% of facilities without water supply). The countries with the largest populations of people impacted by water pollution are Ethiopia (55 million), Nigeria (44 million), Pakistan (43 million), the Democratic Republic of the Congo (30 million), and the Russian Federation (22 million) [126].

**Figure 3:**
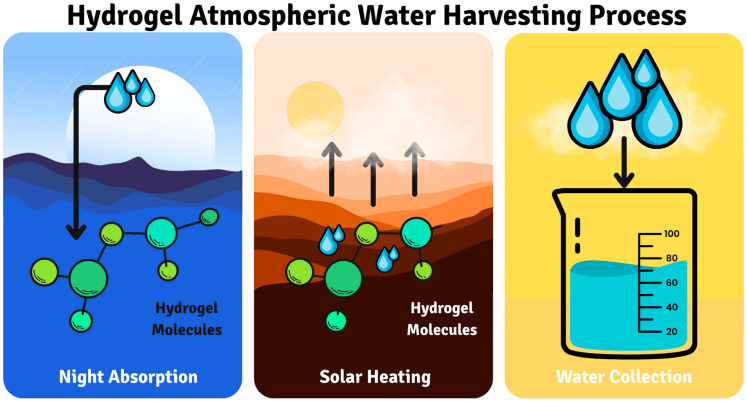
**Hydrogel Atmospheric Water Harvesting Process.** This three-stage process demonstrates hydrogel-based atmospheric water harvesting through: Night Absorption phase where hydrogel molecules absorb water vapor from humid air through hydrogen bonding with hydrophilic functional groups; Solar Heating phase where solar energy heats the hydrogel molecules embedded in soil/substrate to 60–75 °C, releasing the absorbed water; Water Collection phase where released water vapor condenses and is collected. The technology shows particular promise for applications in rural communities, desert regions, emergency relief operations, off-grid systems, and coastal areas. *Figure created using Canva ver. 2025 (accessed on 5 November 2025)*.

Innovative approaches to sustainable water collecting are being developed since water shortage affects billions of people worldwide. Among these, hydrogels are the most promising materials (Figure 3). The composition of hydrogels used in water harvesting applications varies; synthetic hydrogels made mostly of acrylamide (AM), acrylic acid (AA), and its derivatives have good absorption capabilities but raise questions regarding biodegradability [127,128].

The fundamental mechanism of hydrogel-based water harvesting operates through a two-phase sorption-desorption cycle. During the night adsorption phase, hygroscopic polymer networks and incorporated salts (such as CaCl_2_ or LiCl) attract water vapor from humid air through deliquescence, wherein water molecules form hydrogen bonds with hydrophilic functional groups (-OH, -COOH, -NH_2_) on the polymer chains [129,130,131,132]. The 3D porous network structure acts as a water capture matrix, allowing rapid diffusion and storage of water molecules within the gel matrix through osmotic pressure gradients. During the day desorption phase, solar heating (40–80 °C) reverses this process through temperature-induced phase transitions, causing polymer chains to release their bound water molecules as vapor, which condenses on cooled collection surfaces for harvesting. This passive sorption-desorption mechanism requires no external energy input for the absorption phase and minimal thermal energy from solar radiation for the desorption phase, distinguishing it from energy-intensive conventional desalination approaches [7,15].

#### 3.1.1. Advanced Hydrogel Formulations for Water Harvesting

The addition of hygroscopic salts to hydrogel matrices is a significant advancement in water harvesting, as it greatly increases moisture absorption at low humidity levels. In order to overcome challenges like polymer degradation, loss of mechanical strength, and decreased functionality in high-salinity environments, researchers have developed, e.g., salt-friendly polyzwitterionic polymers that effectively bind salt ions while maintaining structural integrity [27,60,133,134]. However, salt incorporation introduces long-term durability concerns, as salt crystallization can cause mechanical stress and reduce efficiency over multiple cycles [135]. Studies show that even advanced systems maintain performance for only 10–24 cycles, far short of the thousands needed for practical deployment [26]. Water can also be efficiently collected from fog droplets at a rate of about 5.0 g/(cm^2^·h) thanks to the porous, hydrophilic micro-tree design of a PVA/PPy hydrogel membrane with a micro-structured surface. With an evaporation rate of 3.64 kg/(m^2^·h) at 1 sun irradiation, the PVA/PPy hydrogel membrane performs two tasks: it effectively captures fog at night and generates interfacial solar steam during the day [136,137,138,139]. These evaporation rates require complex thermal management systems and are achieved only under optimal laboratory conditions [136]. Additionally, at high humidity (~90%), spongy hygroscopic hydrogels have demonstrated rapid water release and vapor uptake in addition to water absorption capacities of about 3.5 g g^−1^ [140]. Because of these properties, these hydrogels are effective at absorbing atmospheric water, especially in humid or foggy environments, and the design demonstrates excellent stability by maintaining both water harvesting functions after more than twenty months of storage, making it a promising technology for sustainable all-day water harvesting [136,141]. Critically, performance degrades substantially at low humidity conditions (<30% RH) where water scarcity is most acute, with no current sorption-based system achieving adequate water production in truly arid environments [27].

#### 3.1.2. Thermo-Responsive Systems

Thermo-responsive hydrogels, an advancement in atmospheric water harvesting, are composed of hygroscopic and antifouling monomers such as vitamin B_5_ analogs, poly(N-isopropyl methacrylamide) (PNIPAM), and natural polymer blends like alginate and ionic salts. These hydrogels allow for controlled water release at moderate temperatures and effective moisture capture [22,84,85,92,142]. Up to 85% of the absorbed water can be released by gently heating these materials to roughly 60 °C thanks to temperature-induced phase transitions. However, thermal-driven release creates a fundamental energy balance challenge, with 50% of absorbed water requiring 30 min of thermal input in “express mode”, followed by 60 min for the remaining 50%, significantly limiting off-grid applications [27]. Because of their antimicrobial qualities, bacteria cannot grow when water is absorbed, making the collected water safer to drink [143]. Recent studies have further improved these hydrogels by incorporating nanostructured additives like metal–organic frameworks (MOFs), which significantly enhance water uptake even under low humidity conditions [144,145]. Additionally, the development of dual-responsive hydrogels that react to both temperature and humidity fluctuations allows for more efficient and adaptive water harvesting cycles [146,147]. These materials also exhibit improved mechanical stability and faster response times, making them more practical for real-world applications. Integration with solar-thermal systems enables these hydrogels to harness sunlight for heating, reducing reliance on electrical energy and making them especially suitable for off-grid and rural environments [148,149]. Nevertheless, the incorporation of advanced additives like MOFs and hygroscopic salts significantly increases manufacturing costs, making these systems economically unviable for addressing basic water needs in developing regions [61].

#### 3.1.3. Performance Analysis and Commercial Viability

Despite the potential of hydrogel-based atmospheric water harvesting for supplying clean water [28,150,151,152,153], its widespread adoption is limited by the high cost of effective hydrogels (especially with advanced features like hygroscopic salts and nanostructures enhancing moisture uptake and stability [154,155]), concerns about the environmental impact of non-biodegradable types, and maintenance in challenging environments. Furthermore, mechanical degradation under repeated swelling-drying cycles poses significant durability concerns, with UV radiation exposure causing severe photooxidative degradation leading to polymer chain breakage and reduced performance over time [156]. Current testing periods span only weeks to months rather than the years required for practical water security applications [157]. Nevertheless, practical examples like SOURCE Global’s solar-powered “hydropanels” in dry areas and pilot programs in rural India demonstrate its capability for independent, low-energy water provision, offering portability and a broad humidity operating range, unlike fog nets (cheaper in foggy areas) and reverse osmosis (preferred for large-scale supply despite high energy use). Combined with eco-friendly production and renewable energy like solar-thermal systems [158,159,160], ongoing research into performance and scalability suggests hydrogels can contribute to water harvesting for a more water-secure future. Nevertheless, the fundamental challenge remains that hydrogel-based systems are most effective in humid coastal regions where alternative water sources are typically available, while showing reduced performance in the arid inland conditions where water scarcity is most acute [28].

Hydrogels have emerged as an innovative technique in the attempt to supply clean water to billions of people worldwide. These materials’ molecular interactions and intelligent responsiveness allow them to efficiently absorb atmospheric moisture and release it when needed, even in harsh environmental conditions [150,151,152]. The use of advanced compounds like hygroscopic salts and nanostructured components, which enhance their moisture uptake and cycling stability, makes them efficient even in arid or low-humidity environments [26,161,162]. When combined with environmentally friendly production techniques and renewable energy sources like solar-thermal systems, hydrogels provide practical and sustainable water harvesting solutions [108,163,164]. However, current limitations include high manufacturing costs for advanced formulations, potential degradation under prolonged UV exposure, and salt crystallization issues that can reduce efficiency over time. Additionally, the water production rates remain relatively low compared to traditional desalination methods, limiting their applicability for large-scale water supply systems. The energy requirements for effective water separation often exceed the available solar thermal input, particularly during cloudy periods or in winter conditions when water demand is highest. Moreover, infrastructure requirements for condensation, thermal management, and water purification add significant operational complexity and maintenance costs that limit deployment in resource-constrained settings [165]. Hydrogels have the potential to change water harvesting technologies as research into their performance and scalability continues, providing potential for a future that is more resilient and water-secure, but overcoming the critical barriers of energy-efficient water separation, long-term material durability, and economic viability for large-scale deployment remains essential for a meaningful contribution to global water security [13].

Hydrogel-based atmospheric water harvesting offers low energy consumption (0.1–0.5 kWh/m^3^) compared to reverse osmosis, but its efficiency plummets significantly at lower humidities, decreasing by 70–80% below 40% relative humidities. Furthermore, current systems are largely at the pilot scale, with commercial options like SOURCE hydropanels facing a substantial cost premium of 5–20× over conventional desalination, severely limiting their broader market penetration. Real-world performance is also hampered by degradation factors such as salt crystallization, UV exposure, dust accumulation, and freeze–thaw cycles, necessitating frequent maintenance. Consequently, while promising for niche or emergency applications, hydrogel-based atmospheric water harvesting is not yet viable for large-scale municipal water supply due to performance limitations, high costs, and operational challenges [166,167].

Advanced hydrogel formulations demonstrate quantified superiority with water adsorption capacities reaching 5.6–6.7 g g^−1^ at 90% RH compared to <1 g g^−1^ for conventional materials [26]. Optimized solar–wind coupling systems achieve 14.9 L/m^2^/day with 25.7% thermal efficiency. High-yield atmospheric water capture via bioinspired material segregation, while PAM-LiCl systems produce 1.7 L/m^2^/day with 16% efficiency in arid conditions like the Atacama Desert [28,29]. Molecular dynamics simulations reveal dual-phase water absorption mechanisms with diffusion coefficients of 1.81–2.97·10^−7^ m^2^/s. Mixed diffusion and relaxation kinetics model for hydrogels swelling, governed by surface tension effects followed by internal polymer network diffusion [168].

The diverse AWH approaches described above exhibit distinct performance profiles optimized for different deployment scenarios. To facilitate system selection and technology assessment, Table 4 consolidates the key performance metrics-operating conditions, water production flux, and regeneration energy-enabling direct comparison with conventional desalination technologies.

### 3.2. Hydrogels as Cooling Agents

The ongoing rise in global temperatures has significantly increased cooling demand in buildings. Hydrogels offer promising materials for thermal management through efficient evaporative cooling mechanisms [121,171]. The need for cooling in residences, workplaces, and commercial buildings has significantly increased as a result of this heat wave [172,173,174]. On the other hand, conventional cooling techniques are energy-intensive and emit large amounts of greenhouse gases, which worsen climate change by generating a negative feedback loop in which rising temperatures increase cooling requirements and emissions [175,176,177].

#### 3.2.1. Cooling Mechanisms and Performance

Hydrogel cooling functions through two complementary synergistic mechanisms. The primary mechanism is evaporative cooling, where water molecules on the hydrogel surface absorb thermal energy equal to the latent heat of vaporization. The hydrogel’s high water content serves as a continuous water reservoir, while its porous hydrophilic matrix maintains a constant supply of water at the surface. Hydrogen bonding between water and polymer chains regulates controlled water release rates, enabling sustained evaporative cooling without electrical input. The secondary mechanism is passive radiative cooling, wherein the hydrogel’s infrared transparency in the atmospheric window allows thermal radiation to escape directly to space. Simultaneously, incorporated light-scattering particles (TiO_2_, SiO_2_) reflect incoming solar radiation, preventing heat absorption. These dual mechanisms work synergistically to achieve temperature reductions below ambient conditions even under direct sunlight, a performance level that mechanical cooling systems cannot accomplish without continuous electrical power input [31,33,178,179].

**Figure 4:**
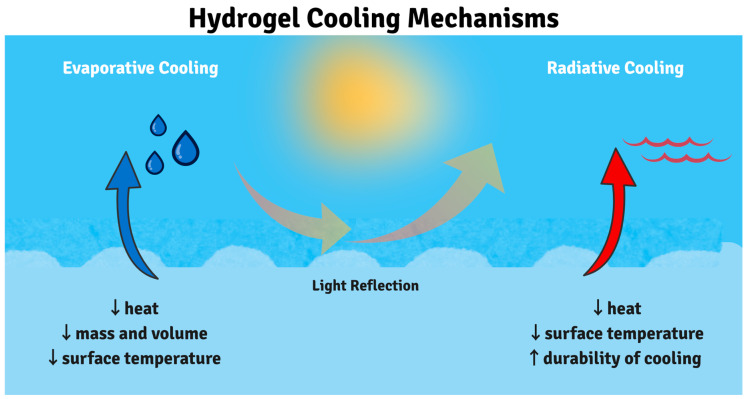
**Hydrogel Passive Cooling Mechanisms and Applications.** Two primary cooling mechanisms are employed by hydrogel systems: Evaporative Cooling (left panel) where water evaporation from the hydrogel surface absorbs latent heat, with high water content enabling sustained cooling that reduces heat, mass/volume, and surface temperature without energy input; and Radiative Cooling where the hydrogel surface reflects solar and emits infrared radiation back to space through the atmospheric window, reducing heat and surface temperature while increasing cooling durability. The hydrogel layer serves as the cooling medium. Applications span personal cooling devices, building materials, electronics cooling, agricultural protection, automotive systems, and outdoor equipment, demonstrating the versatility of passive cooling approaches. *Figure created using Canva ver. 2025 (accessed on 5 November 2025)*.

Hydrogels are promising materials for thermal management, particularly for cooling applications, due to their efficient evaporative cooling mechanism (Figure 4). Their 3D polymer network can retain a high-water content—up to 90%, which serves as the primary reservoir for heat absorption through water evaporation, a process that absorbs significant heat and provides prolonged cooling that outperforms traditional methods [30,34,57,180,181]. The cooling performance of hydrogels can be further enhanced by incorporating hygroscopic substances such as calcium chloride or lithium salts. These additives improve water retention, impart antifreeze properties, and enable self-rehydration from atmospheric moisture, thereby extending the hydrogel’s functional lifespan for sustainable passive cooling. In addition to evaporative cooling, hydrogels also exhibit radiative cooling capabilities by reflecting infrared radiation [30,182]. When combined, evaporative and radiative cooling mechanisms, especially when paired with insulating layers, result in longer cooling durations and greater temperature reductions. Thanks to these synergistic effects, hydrogels are increasingly applied in personal cooling devices and electronics thermal management, achieving temperature reductions, enhancing both efficiency and lifespan. Ultimately, the polymer network structure and crosslinking density of hydrogels are critical factors that balance water absorption capacity, mechanical strength, and thermal properties. This balance ensures stable and efficient cooling performance across a variety of energy-efficient thermal management applications, including building materials, packaging, and agriculture [183,184,185,186,187].

#### 3.2.2. Comparative Analysis with Conventional Cooling

The effectiveness of hydrogel cooling for addressing the broader climate crisis requires careful evaluation when compared to established large-scale cooling methods, particularly conventional evaporative cooling systems that utilize wetted surfaces with controlled water application. While hydrogels can achieve significant local cooling effects with temperature reductions up to 18 °C in controlled conditions as demonstrated by smart window applications, their performance per unit cost and area faces limitations compared to traditional evaporative cooling systems for large-scale applications [7,14,30,180,188]. Evaporative cooling systems are highly energy-efficient alternatives to conventional air conditioning, with studies showing energy savings of 33% during summer and 26% during winter seasons, and some systems consuming less power while saving 30% of energy compared to conventional AC (air conditioning) systems. Research demonstrates that evaporative coolers cost about half as much to install as central air conditioners and use about one-quarter as much energy [189,190,191]. In contrast, hydrogel-based systems, while requiring no direct energy input during operation, face substantial upfront material costs and durability challenges [183,185]. Evaporative cooling systems demonstrate proven cost-effectiveness for large-scale applications, with the technology being one of the cheapest and oldest cooling methods available [192], while specialized hydrogel applications have achieved production costs as low as $3.6 per square meter [53], though advanced cooling formulations require significantly higher investment. Additionally, hydrogel cooling systems require periodic replacement or regeneration due to UV radiation exposure causing severe photooxidative degradation of polymeric materials, which results in polymer chain breakage, free radical formation, and molecular weight reduction leading to significant deterioration of material properties [193].

#### 3.2.3. Commercial Applications and Market Position

Hydrogel-based cooling systems have become more widely used and commercially available in recent years. Passive, electricity-free cooling for comfortable sleep is provided by products such as hydrogel cooling pads and mattress toppers. Hydrogel-infused panels and coatings are being tested in the building industry to lower indoor temperatures and the need for air conditioning, particularly in smart window systems where thermochromic hydrogels provide both cooling and solar heat gain control, achieving heating, ventilation, air conditioning load reductions ranging from 19.1% to 54% depending on climate conditions [86,194,195,196,197]. The potential of hydrogel-based personal cooling wearables to produce quick, reusable cooling effects has also been investigated. These substances absorb and retain water, which causes the skin to feel cooler when it evaporates. For instance, it has been demonstrated that hydrogel-based cooling garments can sustain skin temperature drops for prolonged periods of time following a water soak, making them useful for individual thermal control in extreme heat conditions where conventional cooling is unavailable [198,199]. Additional promising applications include agricultural crop protection during heat waves providing localized cooling zones, electronics thermal management where precise passive cooling is required, and remote or off-grid applications where minimizing energy infrastructure is critical.

Beyond cooling performance, hydrogels offer economic and environmental benefits by operating passively and reducing energy use and carbon emissions compared to conventional air conditioning [31,200,201]. Advances in biodegradable and bio-based hydrogels address disposal concerns, allowing safe breakdown and potential use as soil conditioners [178,202]. Researchers are also exploring smart sensing integration for adaptive thermal management [17,203,204], highlighting hydrogels’ versatility in sustainable cooling solutions. Rather than replacing large-scale cooling infrastructure, hydrogels show greater potential as complementary technologies within integrated cooling strategies, including hybrid systems that combine hydrogel materials with conventional evaporative cooling to enhance efficiency in specific microclimates, peak load reduction during extreme temperature periods to reduce strain on electrical grid-dependent cooling systems, and distributed cooling networks in urban areas as part of heat island mitigation strategies where traditional cooling infrastructure is impractical [30,32]. Despite these advantages, several challenges limit widespread adoption, including limited cooling duration that requires frequent rehydration, potential bacterial growth in water-rich environments, and reduced effectiveness in extremely dry conditions. Furthermore, the cooling capacity may be insufficient for large-scale applications, and mechanical degradation can occur under repeated swelling-drying cycles. Most critically, the economic viability for large-scale climate applications remains questionable, with material and replacement costs significantly exceeding those of conventional evaporative cooling systems, making hydrogel cooling more suitable for niche applications and personal thermal management rather than transformative climate crisis solutions at the scale required for meaningful global impact [53,77].

Hydrogel evaporative cooling offers an energy-free temperature reduction of 5–15 °C, surpassing traditional evaporative coolers but not matching the robust performance of mechanical air conditioning in extreme heat. While initial costs are favorable compared to air conditioning, their cooling duration is limited (2–8 h in low humidity, 30–60 min in high humidity), and they require significant recharge times. Consequently, despite zero operational energy costs, the intermittent nature, performance limitations in hot and humid conditions, and potential annual replacement needs restrict hydrogel cooling to moderate climates and specific intermittent cooling applications [32,34].

Hydrogels can achieve surface temperature reductions of up to 15 °C below ambient solely through evaporative cooling, corresponding to cooling powers in the range of 50–150 W m^−2^ under typical laboratory conditions [30,31]. The incorporation of vertically aligned, microporous structures further enhances water transport and extends continuous cooling durations beyond 8 h at 40% relative humidity, maintaining positive cooling power (~56 W m^−2^ at 20 °C) down to 20 °C. Simultaneously, hygroscopic additives such as CaCl_2_ not only depress the freezing point to −20 °C but also enable self-rehydration from ambient moisture, ensuring rapid performance recovery overnight [30,33,205].

### 3.3. Hydrogels for Soil Health

**Figure 5:**
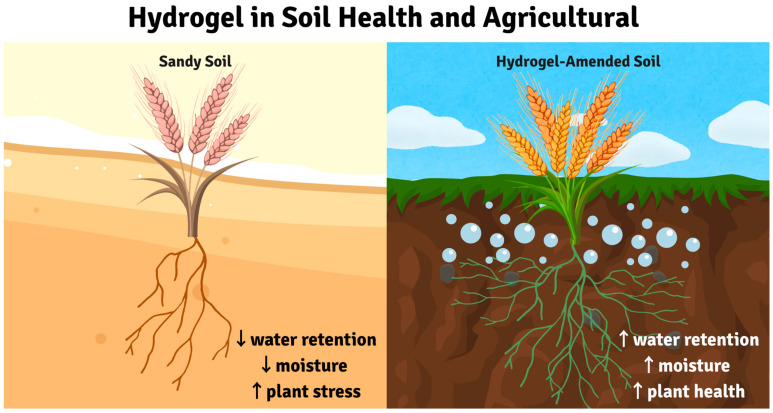
**Hydrogel Enhancement of Soil Health and Agricultural Performance.** Comparative analysis of unamended sandy soil versus hydrogel-amended soil. Unamended sandy soil exhibits characteristic low water-holding capacity, reduced root biomass, and visible drought stress symptoms in plants. Hydrogel-amended soil with hydrogel particles, results in darker soil coloration indicative of elevated hydrometric water content, enhanced plant proliferation. Quantitative improvements in hydrogel-amended systems include increase in soil water retention capacity, reduction in irrigation requirements, crop yield enhancement, and improvement in soil porosity. *Figure created using Canva ver. 2025 (accessed on 5 November 2025)*.

Sandy soils, characterized by poor water retention, are particularly vulnerable to desertification. Superabsorbent hydrogels (SAHs) address this challenge by absorbing and retaining large amounts of water. These materials can absorb and retain large amounts of water, releasing it gradually to plants, thereby improving soil moisture and promoting sustainable agriculture (Figure 5) [206,207,208].

#### 3.3.1. Performance Metrics and Mechanisms

Quantitative analysis reveals hydrogel soil amendments achieve water retention increases up to 50% in sandy soils, with bulk density reductions from 1.46 to 1.27 g/cm^3^ at 0.4% application rates [36,40,41,209]. Nano-silica hydrogels demonstrate maximum rice yields of 10.76 tons/ha with 90% irrigation, while meta-analysis across crops shows yield improvements of 11–51% depending on species [37,210]. Recent research has focused on developing SAHs from sustainable and eco-friendly sources, such as agricultural waste materials. These materials, including wheat straw, rice husk, and corn stover, possess high contents of cellulose, hemicellulose, and lignin, making them suitable for hydrogel synthesis. The use of agro-waste-derived SAHs not only enhances soil properties but also contributes to a circular economy by reducing waste and minimizing the reliance on petroleum-based materials, thus mitigating greenhouse gas emissions [40,41].

One example is the synthesis of an eco-friendly hydrogel composed of borax and locust bean gum, which demonstrates potential for enhancing sandy soil properties. This hydrogel exhibits a high water-absorbing capacity (130.29 g g^−1^) and, when mixed with sandy soil substantially improves key soil parameters. Specifically, the hydrogel increases maximum soil water content by 32.03%, extends water retention time by 14 days, and enhances soil porosity and organic matter content by 38.9% and 8.64 g/kg, respectively. Importantly, the hydrogel shows minimal toxicity towards soil microorganisms and exhibits 43.47% biodegradation after 4 weeks, indicating its low adverse environmental impact. These findings suggest that locust bean gum/borax hydrogels can effectively improve the water retention capacity of sandy soil, offering a promising avenue for supporting plant growth in water-scarce arid environments [42].

Extremely high absorption capacities (>500 g g^−1^) can cause “reverse osmosis effect” during drought, where hydrogels extract moisture from plant roots when water-deprived. The 130.29 g g^−1^ capacity represents an optimal balance-sufficient for water storage while minimizing root dehydration risks. Research indicates 50–200 g g^−1^ as the ideal range for agricultural applications [50,211].

#### 3.3.2. Multifunctional Soil Improvement

The mechanisms by which hydrogels improve soil health are multifaceted. Their high water absorption and retention capabilities directly address the primary limitation of sandy soils. By holding water, hydrogels reduce water loss through evaporation and deep percolation, making it available to plant roots over extended periods. This enhanced water availability improves plant growth, particularly under drought conditions [39,212]. Furthermore, some hydrogels can also be used for the slow release of nutrients, further promoting plant development and reducing the need for frequent fertilizer applications [206]. The improved soil moisture and nutrient availability create a more favorable environment for soil microorganisms, which play a crucial role in nutrient cycling and overall soil health. While the focus is often on water retention, hydrogels can also play a role in the remediation of contaminated soils. For example, studies have explored the use of hydrogels in conjunction with biochar to immobilize heavy metals and polycyclic aromatic hydrocarbons, reducing their bioavailability and mitigating their toxic effects on plants and microorganisms [213]. The combination of hydrogels and biochar can create synergistic effects, where the hydrogel enhances the water retention and physical properties of the soil, while the biochar contributes to pollutant adsorption and long-term stabilization. Huang et al. demonstrated that functional hydrogels can promote vegetable growth in cadmium-contaminated soil, highlighting their potential in mitigating the negative impacts of heavy metal pollution on agriculture [214]. Synthetic polyacrylamide hydrogels offer superior performance but persist in soil for 5–10 years, forming microplastics and potential toxicity concerns. Natural alternatives show lower absorption (20–150 g g^−1^ vs. 300–1000 g g^−1^), reduced durability (10–50 vs. 100+ cycles), and higher costs (2–5×), but biodegrade within 6–18 months. Optimization through ionic crosslinking and natural-synthetic blends (70:30 ratios) can enhance performance while maintaining biodegradability [35,38,50].

#### 3.3.3. Environmental Considerations and Challenges

The development of hydrogels from sustainable resources and their application in agriculture represents a significant advancement in sustainable agriculture. These materials offer a promising solution for improving soil health, enhancing water retention, and promoting plant growth, particularly in water-stressed environments. Further research is needed to optimize the synthesis and application of these hydrogels, and to fully understand their long-term effects on soil ecosystems. Application requires optimization of rates (0.1–0.3% *w*/*w* for sandy soils), quality control for food safety (<0.05% acrylamide), and economic justification varying with water scarcity and crop values. Future priorities include smart pH-responsive systems and long-term environmental impact studies [83]. However, challenges remain regarding the cost-effectiveness of large-scale applications, potential accumulation of non-biodegradable synthetic hydrogels in soil, and the need for standardized protocols for application rates and timing. Additionally, the long-term effects on soil microbiome diversity and potential impacts on groundwater quality require further investigation to ensure sustainable agricultural practices. Hydrogel soil amendments demonstrate consistent water use efficiency improvements (20–40%), yet their impact on crop yields varies greatly and typically achieves break-even points only in high-value crops or severely water-stressed regions. While biodegradable hydrogels offer environmental benefits by enriching soil organic matter and decomposing within 6–18 months, synthetic variants raise concerns about indefinite accumulation and microplastic formation. Competing with established, often more cost-effective solutions like drip irrigation and mulching, hydrogels find their niche primarily in extreme conditions where conventional irrigation is impractical, highlighting a limited, albeit valuable, role in enhancing soil health [215,216].

Having established the mechanisms and performance characteristics of hydrogel-based passive cooling and soil amendments, Table 5 provides a quantitative framework for comparing these applications. The table distinguishes between evaporative and radiative cooling mechanisms while quantifying agricultural water use efficiency across soil types, establishing the metrics referenced in the Introduction and carried throughout this review.

### 3.4. Hydrogels as Energy Saving Particles

**Figure 6:**
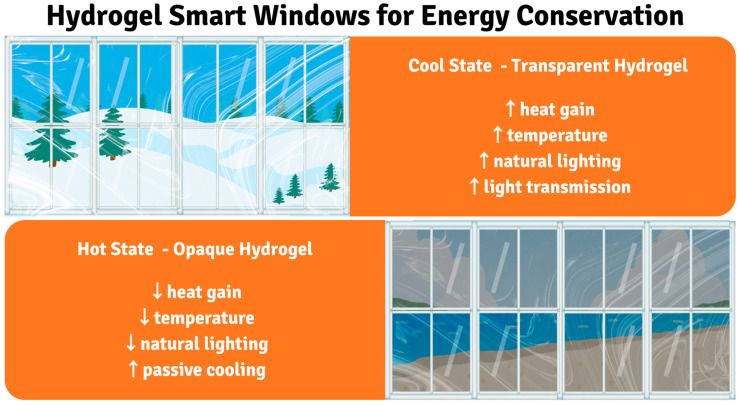
**Thermochromic Hydrogel Smart Windows for Energy Conservation.** Temperature-responsive behavior of hydrogel smart windows displaying two distinct operational states. In cool state—Transparent Hydrogel (below T < 32 °C) with clear window glazing, resulting in increased heat gain, increased temperature, increased natural lighting, and increased light transmission (95% visible light transmittance). The hot state—Opaque Hydrogel (above T > 32 °C) with translucent/frosted window appearance and maintaining diffuse light transmission, resulting in decreased heat gain, decreased temperature, decreased natural lighting, and increased passive cooling through solar modulation. Applications encompass commercial buildings, residential windows, automotive glass, industrial skylights, greenhouse control, and healthcare facilities. *Figure created using Canva ver. 2025 (accessed on 5 November 2025)*.

Hydrogels have emerged as promising materials for energy-saving applications, particularly in smart windows, due to their thermochromic properties and ability to passively regulate indoor environments. These materials offer a compelling alternative to traditional energy-intensive climate control systems by dynamically adjusting light and heat transmission in response to ambient temperature fluctuations [46]. The core principle behind their energy-saving capability lies in their reversible change in transparency with temperature, allowing for effective modulation of solar radiation entering a building (Figure 6).

#### 3.4.1. Operating Principles and Performance

Smart windows using thermochromic hydrogels achieve autonomous energy conservation through temperature-responsive optical modulation without requiring external control systems or electrical input. The fundamental mechanism involves a volume phase transition (VPT) that occurs at the LCST of the polymer, typically around 32 °C for PNIPAm-based hydrogels. Below the LCST, polymer-water hydrogen bonds dominate the intermolecular forces, keeping polymer chains hydrated and extended, allowing the hydrogel to remain transparent with 95% light transmission. Above the LCST, hydrophobic polymer-polymer interactions strengthen and overcome hydrogen bonding, causing polymer chains to collapse and aggregate into a turbid network that scatters visible light, rendering the hydrogel opaque with solar modulation capability. This autonomous switching occurs naturally as ambient temperature changes, requiring zero external power input or sensor systems because temperature sensing is inherent to the polymer’s molecular structure itself. As ambient temperature increases above LCST, the hydrogel automatically transitions from a clear state (allowing solar heat gain and visible daylighting in winter) to an opaque state (blocking unwanted solar radiation in summer), thereby dynamically reducing the building’s energy demand for heating and cooling without active control [44,45,219].

Unlike many conventional smart window technologies that demand external power supplies or complex control mechanisms, thermochromic hydrogel-based systems operate without the need for additional energy input, thereby simplifying their structure and reducing overall energy consumption. This passivity operation is a significant advantage, making them attractive for large-scale architectural integration aimed at improving building energy efficiency [45,46]. The underlying mechanism often involves a phase transition within the hydrogel, such as the volume phase transition exhibited by certain polymer networks. Quantitatively, thermochromic PNIPAm-based hydrogels have demonstrated luminous transmittances (T_lum_) exceeding 95% and solar modulation abilities (ΔT_solar_) up to 33% in prototype smart windows, reducing solar heat gain by approximately 25 W m^−2^ per °C of ambient temperature change [44,220]. The adjustable lower critical solution temperature (LCST) can be tuned between 20 °C and 50 °C by copolymer composition, enabling adaptation to diverse climate zones without external power [44,220].

#### 3.4.2. Alternative Materials and Enhanced Systems

Beyond PNIPAm, other thermosensitive organic materials like hydroxypropyl cellulose (HPC) hydrogels also play a vital role in thermochromic smart windows. The optical performance of these hydrogels can be fine-tuned by adjusting parameters such as film thickness, which influences their transparency and solar modulation ability. Research has shown that hydrogel composites, such as those combining hydrogels with vanadium dioxide (VO_2_), can exhibit enhanced optical properties and maintain good reversibility and performance stability over numerous cycles, provided proper sealing prevents moisture evaporation [48,221]. The Wolde-Kidan et al. discussed particle diffusivity and free-energy profiles in hydrogels in a broader context, including biological systems and drug delivery, highlighting the general understanding of hydrogel properties that underpins these applications. This robust performance under varying thermal conditions underscores their potential for long-term applications in building envelopes [222].

#### 3.4.3. Market Potential and Implementation Challenges

The development of hydrogels as energy-saving materials aligns with the global imperative to reduce energy consumption in buildings, which accounts for a substantial portion of total energy use. By intelligently responding to environmental stimuli without requiring external power, these materials contribute to a more sustainable built environment, offering a simple yet effective solution to mitigate energy loss through windows. The ongoing research into optimizing their performance, cost-effectiveness, and integration methods continues to pave the way for their widespread adoption in future green building initiatives. Their capacity to autonomously manage solar heat gain and natural light makes hydrogels a cornerstone in the evolution of energy-efficient building materials, holding considerable promise for a more eco-friendly world. Nevertheless, current limitations include relatively narrow temperature response ranges that may not suit all climatic conditions, potential optical haze that can affect visual clarity, and durability concerns under prolonged UV exposure and temperature cycling. The scalability of manufacturing processes and integration challenges with existing window systems also present barriers to widespread commercial adoption.

Thermochromic hydrogel windows offer energy-free solar modulation (40–70%), making them a cost-effective alternative to electrochromic systems despite slower response times and less precise optical control. However, their real-world energy savings are limited (15–30% in residential, 5–15% in commercial) and most effective in moderate climates, as performance is compromised in mechanically ventilated buildings or extreme temperatures. Moreover, integration challenges like accelerated degradation (3–5 year lifespan vs. 10–15 theoretical) due to thermal cycling, and maintenance needs like annual resealing, significantly impact their practical viability and long-term cost-effectiveness [43,223].

### 3.5. Comparative Evaluation of Hydrogel Technologies

The positioning of hydrogel technologies within the broader landscape of climate solutions requires critical evaluation against established alternatives to determine optimal deployment strategies (Table 6). Unlike membrane-based desalination technologies that achieve water production through high-pressure filtration requiring 3–4 kWh/m^3^, hydrogel atmospheric water harvesting operates through passive sorption-desorption cycles, eliminating energy penalties but constraining output to 2–5 L/m^2^/day compared to desalination’s 100–1000 L/m^2^/day capacity. This fundamental difference positions hydrogels as complementary rather than replacement technologies for centralized water treatment, excelling in distributed applications where energy infrastructure is limited or environmental impact must be minimized [26,166].

Thermal management applications reveal distinct operational paradigms between hydrogel systems and conventional cooling technologies. Vapor compression air conditioning provides precise temperature control with rapid response times but operates as a closed-loop system requiring continuous electrical input and refrigerant circulation. Hydrogel cooling functions as an open thermodynamic system, exchanging water vapor with the environment to achieve temperature reduction without mechanical components or electrical consumption. This distinction makes hydrogel systems particularly suitable for applications requiring passive operation, such as agricultural crop protection, outdoor worker cooling, or emergency thermal management where grid power is unavailable [34,224].

In agricultural soil conditioning, hydrogel amendments demonstrate fundamentally different water management principles compared to irrigation infrastructure and surface mulching. Drip irrigation systems deliver water directly to plant root zones with 85–95% efficiency but require extensive piping networks, filtration systems, and pressurization equipment. Mulching provides evaporation suppression through physical barriers but lacks the dynamic water storage and release capabilities inherent to hydrogel matrices. Hydrogels function as distributed water reservoirs within the soil profile, absorbing excess moisture during irrigation or precipitation events and releasing it gradually as soil moisture depletes, creating a buffering effect that neither irrigation systems nor mulching can replicate [215,218].

Building energy management through thermochromic hydrogel windows operates on autonomous optical modulation principles distinct from active smart glass technologies. Electrochromic windows achieve precise optical control through applied voltage but require electrical infrastructure, control systems, and ongoing power consumption. Hydrogel-based smart windows respond directly to ambient temperature changes without external control, making them inherently fail-safe and maintenance-free but limiting control precision. This autonomous operation proves advantageous in applications where electrical complexity must be minimized or where fail-safe operation is critical, such as in remote buildings or developing regions with unreliable power infrastructure [43].

Hydrogel technologies demonstrate distinct performance profiles and deployment potential across atmospheric water harvesting, passive cooling, and soil amendment applications, each contributing uniquely to climate adaptation strategies. Atmospheric water harvesting offers sustainable freshwater production with low energy input but remains limited by relatively low water yield (2–5 L m^2^/day) and dependence on humidity levels above 30%; high initial material costs and durability issues constrain widespread adoption, confining current use to niche or off-grid regions [26,28,166,167]. Passive cooling through hydrogels provides energy-free temperature reductions up to 15 °C, with cooling power up to 150 W/m^2^; however, intermittent cooling duration (2–8 h), rehydration needs, and material degradation under UV exposure limit scalability to moderate climates and specific intermittent applications [30,31,53,77,178]. In contrast, soil amendments with superabsorbent hydrogels offer scalable benefits by improving water retention and reducing irrigation by 30–50%, enhancing crop yields particularly in drought-stressed regions; these integrate more seamlessly with agricultural practices and demonstrate moderate durability though largely applicable in high-value or water-scarce cropping systems [36,209,215]. Overall, while soil hydrogel applications show broader economic feasibility and easier integration with existing systems, atmospheric water harvesting and passive cooling remain challenged by higher costs, operational complexity, and durability concerns, requiring further advances for large-scale deployment. Synergistic integration of these applications could maximize hydrogel impact on climate resilience and sustainable resource management.

**Table 6:** **Climate Applications and Performance Metrics of Hydrogel Technologies.** Performance metrics and commercial status of hydrogel-based climate technologies across six application domains. Key metrics include water production rates (2.4–5.0 L/kg·day), temperature reduction (5–15 °C), and energy savings (up to 45%). Operating conditions span temperature ranges (−10 to 90 °C), humidity requirements (20–90% RH), and pH specifications (4–9).

Application Domain	Hydrogel System	Key Performance Metrics	Operating Conditions	Advantages	Current Limitations	References
**Water** **Harvesting**	Polyelectrolyte hydrogels with hygroscopic salts	2.4–5.0 L/kg·day water output, 85% release efficiency	20–90% humidity, 20–60 °C	Energy-free operation, works in arid climates	High cost, salt leaching, limited scalability	[15,27,28,154]
**Passive** **Cooling**	PVA/hygroscopic salt composites	5–15 °C temperature reduction, 3–8 h cooling duration	40–80% humidity, 25–45 °C	No electricity required, reusable, portable	Limited cooling duration, bacterial growth risk	[30,32,136,178]
**Soil Health**	Superabsorbent agricultural hydrogels	30–50% irrigation reduction, 130–1000 g g^−1^ absorption	pH 6–8, 15–35 °C soil temperature	Drought resistance, nutrient retention, yield increase	Cost-effectiveness, long-term soil effects	[19,36,215,225]
**Energy Saving**	Thermochromic PNIPAM/HPC systems	45% energy reduction, 32 °C transition temperature	Building integration −10 to 50 °C; passive operation without external control	Autonomous operation, high transparency, durability	Narrow response range, UV degradation	[44,47]
**Carbon** **Capture**	PEI hydrogel particles	6.5 mol CO_2_/kg (pure), 0.7 mol CO_2_/kg (air)	25 °C; 400–100,000 ppm CO_2_ range	High selectivity, regenerable, scalable	Regeneration energy, humidity sensitivity	[13,157]
**Environmental** **Remediation**	Polyelectrolyte hydrogels with hygroscopic salts	90–99% heavy metal removal, pH 4–9 operation	Various contaminated environments	Selective adsorption, eco-friendly, solar-powered	Treatment capacity, fouling resistance	[158,159,213,214]

The integration potential of hydrogel technologies with existing infrastructure reveals unique advantages not available with conventional alternatives. Unlike centralized water treatment facilities that require dedicated infrastructure and skilled operators, hydrogel atmospheric water harvesting systems can be deployed incrementally, scaling from individual household units to neighborhood clusters without requiring fundamental infrastructure changes. Similarly, hydrogel soil amendments integrate seamlessly with existing farming practices without necessitating new irrigation infrastructure or changes to planting patterns, unlike precision agriculture systems that require sensor networks and automated control systems [28,166,226].

Environmental impact assessment demonstrates that hydrogel technologies offer distinct sustainability profiles compared to conventional alternatives. Reverse osmosis desalination produces concentrated brine waste requiring disposal or treatment, while hydrogel water harvesting generates no waste streams beyond eventual biodegradation of natural polymer matrices. Refrigerant-based cooling systems create long-term environmental liabilities through ozone depletion potential and global warming potential of synthetic refrigerants, whereas hydrogel cooling relies entirely on water evaporation with no chemical emissions or environmental persistence concerns [227,228].

Economic deployment strategies reveal different optimization approaches for hydrogel technologies versus conventional systems. Traditional infrastructure investments in desalination plants or irrigation systems achieve economies of scale through centralized, high-capacity installations but require substantial capital investment and long payback periods. Hydrogel technologies optimize through distributed deployment and modular scaling, enabling lower initial investment thresholds and shorter payback periods but potentially higher per-unit costs at large scales. This economic structure makes hydrogel solutions particularly attractive for small-scale implementations, emergency applications, and markets where capital availability is limited [8,229,230].

The operational flexibility inherent to hydrogel technologies provides advantages in rapidly changing climate conditions that conventional systems cannot match. Fixed infrastructure systems designed for specific climate parameters may become suboptimal as regional conditions shift due to climate change. Hydrogel systems can be reformulated, redeployed, or adapted to changing conditions through relatively simple material modifications, providing resilience advantages particularly important for long-term climate adaptation strategies where future conditions remain uncertain [231].

## 4. Overcoming Climate Challenges

The increasing climate crisis poses serious threats to human and environmental systems, with compounding issues including growing urban heat islands, prevalent water shortages, and declining soil health necessitating creative and sustainable technical solutions [232,233,234]. Hydrogels have emerged as groundbreaking materials that can address multiple sides of this crisis due to their remarkable water retention, responsiveness, and biocompatibility [166,235,236].

### 4.1. Eco-Friendly Hydrogel Innovations

Thanks to their ability to address several environmental issues at once, hydrogels have quickly progressed to crucial components in the worldwide response to climate change. Their large-volume water-holding 3D networks provide applications beyond cooling, including water collection, soil health improvement, and energy efficiency [237,238,239].

Hydrogels are being used more and more in large-scale infrastructure projects as well as in our daily lives to address important global issues including water shortage and the need for robust agriculture. Innovative atmospheric water harvesters that use hydrogel technology are used as practical solutions in areas with ongoing water shortages [240,241]. Hydrogels are being used increasingly in large-scale infrastructure projects and daily life, including solar-powered hydrogel panels in China and the Middle East directly drawing drinkable water from humid air [242], commercial solutions like Stockosorb^®^ (Evonik, Germany) and Aquasorb^®^ (Jacobi Carbons/Jacobi Group, Premnitz Germany, Columbus, OH, USA) significantly increasing soil matrix water retention in dry and semi-arid locations [35,36,243,244], and green roofs and wall panels with hydrogel being researched to counteract urban heat island effects in densely populated places like Singapore and Los Angeles [245]. An important step in reducing urban energy consumption and the related greenhouse gas emissions is the incorporation of hydrogels into advanced building materials for improved thermal regulation, which lowers indoor temperatures and reduces the need for energy-intensive air conditioning systems [246,247].

The development of biodegradable and bio-based hydrogels (e.g., cellulose or alginate) ensures the environmental sustainability of these climate solutions, allowing for safe decomposition or even soil enrichment in a circular approach [248,249]. This, alongside their diverse applications, from water harvesting and agricultural resilience to urban cooling and sustainable construction, demonstrates hydrogels’ integrative role in climate adaptation and mitigation, leading to their increasing adoption at community and municipal levels and highlighting their versatility and growing impact.

Economic analysis shows hydrogel AWH systems face 5–20× cost premiums over conventional desalination, limiting viability to distributed applications [8]. Agricultural applications achieve positive returns primarily in high-value crops or water-stressed regions, with irrigation reductions of 30–50% offsetting material costs [36].

### 4.2. Synergistic Approaches

By integrating hydrogel amendments with complementary technologies, multiple climate benefits accrue: combining superabsorbent hydrogels with drip irrigation increases irrigation efficiency from 90% to 97% while reducing water application by 40% in field trials, and coupling thermochromic hydrogel window coatings with solar-driven ventilation yields an additional 1.5 °C indoor temperature drop compared to either technology alone in office-scale test cells [223,231]. In Singapore, deploying hydrogel-cooled photovoltaic panels achieved an average module temperature reduction of 10 °C and a corresponding 6% increase in electrical output over a 12-month monitoring period [26]. Such hybrid systems illustrate that strategic pairing of hydrogel solutions enhances performance beyond isolated applications, optimizing resource use and energy savings.

Hydrogel-based solutions offer a powerful, interconnected approach to tackling diverse environmental challenges. When used strategically across different sectors, their impact is amplified, building greater resilience against climate change [250]. For example, hydrogels can be used in agriculture to retain soil moisture and reduce the need for energy-intensive irrigation, which lowers emissions and farmer costs. Large farms in regions like California and Spain have successfully used hydrogels to save water and improve soil health. Combining hydrogels with other sustainable methods like drip irrigation and organic mulching further enhances these positive outcomes [8,87,251].

Integrating hydrogel technologies is reshaping urban planning through initiatives like New York City’s “Cool Roofs”, where hydrogel coatings cool buildings and reduce energy demand during heatwaves. Additionally, hydrogel-based water harvesting systems on rooftops and in public spaces are collecting atmospheric moisture for irrigation and sanitation, enabling the strain on public water resources [252]. Beyond urban applications, hydrogels are being explored for energy systems, with researchers in Japan and South Korea developing them to store thermal energy from solar panels, thereby enhancing renewable energy efficiency [253,254,255]. Importantly, the adaptability of hydrogels allows them to be tailored to specific local needs and integrated into diverse infrastructures, from rural agriculture to urban buildings and remote communities. By fostering collaboration among scientists, engineers, policymakers, and local stakeholders, hydrogel technologies are positioned to become a fundamental component of global sustainable climate adaptation and mitigation strategies [36,256,257].

### 4.3. Integration and Feasability

The successful deployment of hydrogel technologies for climate change mitigation requires comprehensive assessment of their life cycle impacts, operational durability, economic viability, and alignment with global sustainability frameworks. This section addresses critical considerations for decision-makers implementing hydrogel-based climate solutions.

Life cycle assessment studies of hydrogel materials reveal significant variations in environmental impact based on polymer source and synthesis methods. Natural polymer hydrogels from agricultural waste demonstrate carbon-negative potential when accounting for biomass sequestration and waste diversion, with net carbon emissions ranging from −0.5 to 0.2 kg CO_2_-eq per kg of hydrogel produced [50,206]. Synthetic hydrogels exhibit higher carbon footprints (2–5 kg CO_2_-eq/kg) due to petrochemical feedstocks and energy-intensive polymerization processes, though their extended operational lifetimes partially offset initial emissions through sustained performance benefits [23,38]. Energy payback analysis indicates that hydrogel-based atmospheric water harvesting systems achieve positive energy returns within 6–12 months when displacing conventional desalination, with cumulative energy savings of 15–25 MWh over a 5-year operational period [24,25,166].

Field deployment exposes hydrogels to multiple degradation mechanisms that significantly impact operational longevity. UV radiation causes polymer chain scission and crosslink breakdown, with unprotected hydrogels experiencing 40–60% mechanical property loss after 1000 h of exposure (equivalent to 3–4 months outdoor service). UV stabilizers and surface coatings extend service life to 2–3 years, though performance gradually declines [77]. Salt fog exposure in coastal environments accelerates degradation through osmotic stress and crystallization damage, particularly affecting hygroscopic salt-containing formulations. Dust accumulation reduces optical properties by 15–30% in atmospheric water harvesting applications, necessitating periodic cleaning or self-cleaning surface modifications [29,50,154]. Temperature cycling (−10 to 50 °C) induces fatigue through repeated expansion-contraction cycles, with freeze–thaw damage particularly problematic for water-saturated hydrogels [168].

Cyclic performance testing reveals critical thresholds for different hydrogel applications. Atmospheric water harvesting systems maintain 80% of initial water uptake capacity after 100 cycles, declining to 60–70% after 500 cycles due to salt migration and pore collapse [15,154]. Regeneration temperatures significantly impact longevity: operation at 60 °C preserves performance through 500+ cycles, while 80 °C regeneration causes accelerated degradation after 200–300 cycles [60,61]. Passive cooling applications demonstrate superior cycle stability (1000+ cycles) as lower temperature variations minimize thermal stress [30,31]. Agricultural hydrogels undergo 50–100 hydration-dehydration cycles per growing season, with biodegradable formulations intentionally designed for controlled degradation over 6–18 months [19,36]. Optimization strategies including gradient crosslinking, sacrificial bonds, and self-healing mechanisms extend operational cycles by 2–3× while maintaining 90% performance retention [66].

Current hydrogel production costs vary significantly based on materials and scale. Laboratory-scale synthesis ranges from $50–200/kg for natural polymer hydrogels to $20–100/kg for synthetic variants, with costs decreasing by 60–80% at industrial scales (>1000 kg batches) [8,36,167]. Atmospheric water harvesting installations require capital investments of $500–2000 per daily liter of water production capacity, comparing favorably with small-scale desalination ($1000–3000/L/day) when considering energy costs [15,24]. Agricultural hydrogel applications cost $200–500/hectare for initial treatment, providing economic returns through 20–40% water savings and 15–30% yield improvements over 2–3 growing seasons [19,36]. Smart window implementations add $50–150/m^2^ to glazing costs but generate payback periods of 3–7 years through energy savings in commercial buildings [43,44]. Total cost of ownership analyses incorporating maintenance, replacement, and disposal reveal hydrogel solutions achieve cost parity with conventional technologies within 2–5 years for most applications [22,167].

Hydrogel technologies directly contribute to multiple UN Sustainable Development Goals (SDGs), providing measurable impacts across interconnected sustainability challenges. SDG 6 (Clean Water and Sanitation) benefits through atmospheric water harvesting systems producing 2–5 L/day per square meter of collector area, potentially providing drinking water for 1 billion people in water-stressed regions [15,28,166]. SDG 7 (Affordable and Clean Energy) advances through passive cooling technologies reducing building energy consumption by 15–45%, equivalent to avoiding 2–5 tons CO_2_ emissions annually per building [31,43,166]. SDG 11 (Sustainable Cities and Communities) incorporates hydrogel-based green infrastructure for urban cooling, reducing heat island effects by 2–5 °C while managing stormwater through enhanced soil water retention [30,32,36]. SDG 13 (Climate Action) realizes direct mitigation through reduced energy consumption and adaptation through improved agricultural resilience, with hydrogel-amended soils maintaining crop productivity under 30–50% reduced irrigation [19,215].

Policy frameworks increasingly recognize hydrogel technologies within climate adaptation strategies. National adaptation plans in 15 countries explicitly include atmospheric water harvesting as drought resilience measures, with subsidies ranging from 30 to 70% of installation costs [29,167]. Agricultural support programs in water-scarce regions provide hydrogel amendments at reduced costs to smallholder farmers, improving food security while reducing groundwater extraction [8,36]. Building codes in several jurisdictions now incentivize passive cooling technologies through expedited permits and tax credits, accelerating market adoption of thermochromic windows and hydrogel cooling systems [43,44]. International climate finance mechanisms, including the Green Climate Fund, have allocated $500 million for hydrogel-based adaptation projects in developing countries, demonstrating growing institutional support for these technologies [166,167].

### 4.4. Economic Feasibility and Sustainability Assessment

Hydrogel-based climate solutions’ economic feasibility differs greatly depending on the application domain. At industrial scales, production costs drop by 60–80% from $50–200/kg for natural polymer hydrogels to $20–100/kg for synthetic versions [8,36,167]. Agricultural soil amendments cost $200–500/hectare, atmospheric water collection costs $500–2000 per daily liter capacity, and smart windows cost $50–150/m^2^ [15,19,24,36,44]. Water harvesting is comparable to small-scale desalination ($1000–3000/L/day), smart windows produce 3–7 year payback times in commercial buildings, and agricultural applications yield financial returns through 20–40% water savings over two to three growth seasons [19,24,36,43,44]. For the majority of applications, hydrogel solutions reach cost parity with traditional technologies in two to five years, according to total cost evaluations [22,167].

When biomass sequestration and waste diversion are taken into consideration, life-cycle assessments show that natural polymer hydrogels derived from agricultural waste exhibit carbon-negative potential (−0.5 to 0.2 kg CO_2_-eq/kg) [50,206]. Because they use petrochemical feedstocks, synthetic hydrogels have larger carbon footprints (2–5 kg CO_2_-eq/kg), yet their longer operational lifetimes somewhat offset emissions [23,38]. With cumulative savings of 15–25 MWh over five years, hydrogel-based atmospheric water harvesting produces positive energy returns in 6–12 months [24,25,166]. While natural alternatives biodegrade in 6–18 months, enhancing soil organic carbon, synthetic polymers linger in soil for 5–10 years, raising concerns about their accumulation [38,50,77].

Applications of hydrogel support several Sustainable Development Goals. In water- stressed regions, atmospheric water collecting yields 2–5 L/day per square meter, contributing to SDG 6 Clean Water [15,28,166]. Passive cooling helps achieve SDG 7 Affordable Energy by cutting building consumption by 15–45% and preventing 2–5 tons of CO_2_ per building each year [31,43,166]. In order to manage stormwater and reduce heat island effects by 2–5 °C, SDG 11 Sustainable Cities integrates hydrogel infrastructure for urban cooling [30,32,36]. With hydrogel-amended soils that retain production under 30–50% less watering, SDG 13 Climate Action achieves adaptation and mitigation [19,215]. Bio-based hydrogels that value agricultural waste contribute to SDG 12 Responsible Production [40,41,50,206]. SDG 15 Biodegradable soil additions increase life on land, although synthetic versions need to be monitored for microplastic buildup [38,50,258].

Multiple obstacles to implementation include limited operational lifetimes due to UV degradation and environmental stressors that reduce service life to months or a few years [77], performance sensitivity with water harvesting efficiency declining 70–80% below 40% relative humidity [259], and cost increases of 5–20× over conventional solutions [8,166,167]. Lack of uniform procedures and building rules is one example of a policy gap [36,37].

Interventions need to be coordinated. Durability enhancements, scalable production, and biodegradable formulations are research priority [35,38,50]. The Green Climate Fund has allocated $500 million for hydrogel-based adaptation projects, 15 countries have implemented subsidies of 30–70% for atmospheric water harvesting, agricultural support programs offer lower-cost amendments, and building codes encourage passive cooling through expedited permits and tax credits [8,29,36,43,44,166,167]. Quality control could be possible by creating performance testing standards and food safety regulations [36,37]. Responsible implementation can be guaranteed by environmental protections that require for life-cycle assessments and monitoring [38,206].

Realistic expectations regarding constraints, strategic deployment where advantages match local needs, and policy frameworks that strike a balance between innovation and conservatism are all necessary for success. Significant contributions to climate mitigation and adaptation are made possible by concentrating on material durability, cost reduction through bio-based feedstocks from agricultural waste exhibiting carbon-negative potential [40,41,50,206], and performance optimization for difficult situations. While fully biodegradable formulations guarantee environmental friendliness, with natural polymers breaking down in 6–18 months as opposed to 5–10 years for synthetics [50,110], advanced manufacturing techniques like 3D printing and electrospinning offer tailored solutions [72,74].

## 5. Current Limitations and Future Prospects

### 5.1. Key Issues

− Performance Limitations: Water harvesting efficiency decreases 70–80% below 40% relative humidity [259]. Cooling systems require frequent rehydration and face bacterial growth risks in water-rich environments.
− Durability Concerns: UV exposure causes rapid polymer chain degradation, with hydrogel lifetimes under continuous environmental exposure ranging from 6 to 24 months [77,109]. Repeated swelling-drying cycles reduce gel strength by up to 80% over a single season.
− Environmental Impact: Non-biodegradable synthetic hydrogels persist in soil for years and may release toxic monomers. Manufacturing processes involve potentially harmful chemicals requiring careful handling [260].
− Economic Barriers: Advanced hydrogel formulations face cost premiums of 5–20× over conventional alternatives, limiting large-scale deployment [8,166,167].

### 5.2. Comparative Performance Analysis and Research Gaps

Although hydrogel-based climate solutions show clear advantages over traditional technologies, their implementation necessitates careful evaluation of operational limitations and performance trade-offs. Hydrogel-based atmospheric water harvesting yields much lower absolute production rates of 2–5 L/m^2^/day compared to 100–1000 L/m^2^/day for conventional systems, but it achieves greater energy efficiency of 0.1–0.5 kWh/m^3^ compared to reverse osmosis desalination at 3–4 kWh/m^3^. Deployment is restricted to coastal and humid areas due to this performance’s significant reliance on ambient humidity levels above 30% RH [24,25]. Although advanced polyelectrolyte formulations have reached up to 2410 mL/kg/day, this is still insufficient for large-scale municipal supply, limiting off-grid applications and emergency water provision’s economic feasibility [15].

In contrast to traditional air conditioning systems that need 2–5 kW for comparable cooling loads, hydrogel evaporative and radiative mechanisms in passive cooling applications produce 5–15 °C temperature decreases without electrical input. However, radiative methods that achieve sub-ambient cooling of 3–7 °C require clear sky conditions, whereas evaporative systems that supply cooling power up to 150 W/m^2^ become useless in humid areas [30,31,33]. Hydrogels are positioned as complementing rather than replacement solutions for harsh climates due to these cooling capacity constraints [34]. Although they add complexity and cost, recent hybrid radiative-evaporative systems show promise for weather-insensitive performance [30,33].

Applications in agriculture show clear trade-offs between sustainability and performance. With improved water absorption of 300–1000 g g^−1^ and cycling durability of more than 100 cycles, synthetic polyacrylamide hydrogels can reduce irrigation by 30–50% and increase yield by 15–30% [18,19,36,39]. But these materials linger in soil for five to ten years, which raises questions concerning the production of microplastics [38,77]. Although natural substitutes biodegrade in 6–18 months, they have poorer absorption (20–150 g g^−1^), shorter durability (10–50 cycles), and higher costs (2–5 times) than synthetic alternatives [35,50]. According to economic study, agricultural hydrogel applications have an initial treatment cost of $200–500/hectare and yield positive returns through water savings of 20–40% over two to three growing seasons, mainly in high-value crops or water-stressed areas [19,36].

Through passive optical modulation at lower critical solution temperature transitions, usually around 32 °C, thermochromic hydrogel smart windows achieve building energy savings of 15–45% while maintaining greater than 95% luminous transmittance below LCST and blocking solar radiation above this threshold [31,43,44,166]. In commercial buildings, zero-power autonomous operation reduces control system complexity and has payback periods of three to seven years. However, after up to seven years of use, seal failures and water loss happen, and aesthetic issues with color appearance in the transparent state continue [43,44].

Every application domain has critical research gaps. Unprotected hydrogels lose 40–60% of their mechanical properties after 1000 h of UV exposure, or three to four months of outdoor service, while UV stabilizers extend service life to two to three years [77]. Quantitative lifetime predictions under UV exposure and thermal cycling are still poorly characterized. Performance comparison and quality control between studies are made difficult by the lack of standardized assessment procedures [36,37]. Deployment choices are limited by inadequate techno-economic analyses that take servicing requirements, end-of-life disposal, and total cost of ownership into account [22,167]. Long-term monitoring procedures are necessary for prospective environmental fate studies that look at groundwater quality effects, microplastic production kinetics, and soil microbiome implications [38]. Interdisciplinary optimization is required for system integration approaches that combine hydrogels with complementary technologies like drip irrigation and solar-thermal coupling. Field tests have shown the possibility of increasing irrigation efficiency from 90% to 97% while lowering water application by 40% [231]. For reliable deployment forecasts, performance characterization under extreme conditions, such as humidity below 30% and thermal cycling, is still insufficient [259].

### 5.3. Future Research Directions

Despite current limitations in durability, cost-effectiveness, and scalability, hydrogel technologies hold substantial potential for addressing climate challenges through continued innovation and strategic development. Emerging research directions focus on overcoming these barriers while expanding the functionality and practical applicability of hydrogel-based climate solutions.

Promising developments include:

− Smart Materials: Development of multi-responsive hydrogels with AI integration for predictive behavior and self-healing capabilities;
− Sustainable Manufacturing: Large-scale production using bio-manufacturing and agricultural waste feedstocks;
− Integrated Systems: Internet of Things-enabled smart networks for real-time climate monitoring and automated optimization;
− Environmental Solutions: Complete biodegradability with carbon-negative manufacturing processes.

Advanced fabrication techniques including 3D printing and electrospinning enable precise control over hydrogel architecture and functionality, offering opportunities for customized solutions tailored to specific climate applications [72,74]. Integration of metal–organic frameworks and nanostructured additives shows promise for enhancing water harvesting performance even under low humidity conditions, addressing current limitations in arid environments [144,145]. Machine learning approaches demonstrate specificity of 88.4% and sensitivity of 86.2% in predicting hydrogel properties, revolutionizing material discovery and enabling rapid optimization of complex multi-component systems [120]. Furthermore, hybrid systems combining hydrogels with complementary technologies such as solar-thermal coupling and drip irrigation achieve synergistic benefits, with field trials demonstrating irrigation efficiency increases from 90% to 97% while reducing water application by 40% [231]. The development of completely biodegradable formulations from renewable sources ensures environmental compatibility while supporting circular economy principles, with natural polymer hydrogels decomposing within 6–18 months compared to 5–10 years for synthetic variants [50,110].

## 6. Conclusions

Hydrogels have demonstrated remarkable potential as multifunctional materials for addressing climate change challenges. Their unique combination of water absorption capacity, stimuli-responsive behavior, and biocompatibility positions them as versatile solutions for water harvesting, passive cooling, soil health improvement, and energy conservation.

In water harvesting applications, hydrogels offer sustainable approaches to freshwater production through efficient atmospheric moisture capture. For cooling applications, they provide energy-free temperature reduction through evaporative and radiative mechanisms. In agriculture, they address soil health challenges by improving water retention and creating favorable environments for plant growth. As energy-saving materials, thermochromic hydrogels enable passive building climate control [7,144,261].

For cooling applications, hydrogels provide efficient passive cooling through evaporative and radiative mechanisms, reducing the energy demand for conventional air conditioning systems [30,31,146]. Their high-water content serves as a primary reservoir for heat absorption through water evaporation, delivering prolonged cooling effects that outperform traditional methods [32,34]. When integrated into building materials, personal cooling devices, and electronics thermal management systems, hydrogels contribute significantly to energy conservation and carbon emission reduction [7,186,239].

In agriculture, hydrogels address soil health challenges by improving water retention in sandy soils, reducing water loss through evaporation and deep percolation, and creating favorable environments for soil microorganisms [36]. Biodegradable hydrogels derived from sustainable sources, such as agricultural waste materials, not only enhance soil properties but also contribute to a circular economy by reducing waste and minimizing reliance on petroleum-based materials [125,206,258].

As energy-saving materials, hydrogels in thermochromic smart windows passively regulate indoor environments by dynamically adjusting light and heat transmission in response to ambient temperature fluctuations [44,46]. Their ability to operate without external power input simplifies their structure and reduces overall energy consumption, making them attractive for large-scale architectural integration [43,262].

The ongoing development of biodegradable and bio-based hydrogels ensures environmental sustainability. When deployed strategically across different sectors, hydrogel-based solutions offer an interconnected approach to building climate resilience. Future research should focus on optimizing hydrogel properties, enhancing durability, and developing cost-effective manufacturing processes. Integration with other sustainable technologies will maximize their impact on climate change mitigation and adaptation strategies. Through continued innovation and strategic deployment, hydrogels represent promising materials that can contribute to creating more sustainable and resilient solutions to environmental challenges.

In conclusion, hydrogel-based climate solutions present a multifaceted approach with promising applications in water harvesting, passive cooling, and soil enhancement. Realizing their full potential requires prioritizing material durability improvements to withstand environmental stressors such as UV radiation and thermal cycling, alongside scalability through sustainable, low-cost manufacturing processes. Integration with existing infrastructure and complementary technologies will enhance their effectiveness and adoption. Policy frameworks that incentivize research, deployment, and commercialization—through subsidies, inclusion in climate adaptation plans, and standards development—are essential to overcome economic and operational barriers. Furthermore, implementing standardized life cycle assessments and long-term field evaluations will ensure environmental safety and cost-effectiveness. Collectively, these design and policy strategies will accelerate hydrogel technology translation from laboratory innovation to impactful climate adaptation tools that enhance resilience, conserve resources, and promote sustainable agricultural and urban environments [263,264,265].

## Figures and Tables

**Table 3:** **Comparative Performance Metrics for Synthetic Hydrogels in Climate Applications.** Synthetic hydrogels offer enhanced water harvesting, CO_2_ capture, and passive cooling capabilities under varying humidity, temperature, or environmental conditions. Their structure and additives help retain water, reduce irrigation needs, and enable efficient climate adaptation strategies in agriculture and atmospheric applications.

Hydrogel Type	Application	Performance Metric	Test Conditions	Reference
Polyelectrolyte	Atmospheric Water Harvesting	2410 mL/kg/day	30% RH, 25 °C, 3 h cycles (8 cycles/day)	[15]
Polyacrylamide-based	Atmospheric Water Harvesting	2.76 g g^−1^ water uptake	90% RH, 25 °C, 24 h absorption	[84]
Salt-embedded composite	Atmospheric Water Harvesting	0.8–1.5 g g^−1^ at low RH	<30% RH, 25 °C, diurnal cycles	[85]
PEI hydrogel particles	CO_2_ Capture (pure)	6.5 mol/kg	100% CO_2_, 25 °C, 1 bar, 30 min cycles	[13]
Hygroscopic salt-polymer	Water Harvesting (desert)	~5.6 g g^−1^ max capacity	Enhanced with LiCl/CaCl_2_	[85]
Thermochromic composite	Passive Cooling	22 °C reduction	Ambient conditions, solar exposure, summer outdoor testing	[86]
PAAm-based	Agricultural water retention	30–50% irrigation reduction	Field studies, maize and soybean-wheat systems	[8,87]

**Table 4:** **Atmospheric water harvesting performance metrics.** Quantitative comparison of hydrogel-based AWH systems showing operating conditions (RH, temperature), water production flux, and regeneration energy requirements. Values represent typical performance under standard test conditions. Conventional reverse osmosis (RO) shown for reference.

Hydrogel System	RH (%)	Temperature (°C)	Water Flux (L/m^2^/day)	Regeneration Energy Requirements	Key Feature	References
PNIPAm-salt composite	90	25–40	5.6–6.7	0.3–0.5	High capacity	[26,29,167]
Solar-wind coupled	60–80	20–35	14.9	0.1–0.2	Highest yield	[28,29,167]
Alginate-CaCl_2_	40–70	15–30	2.4–3.8	0.4–0.6	Low-RH capable	[169]
MOF-hydrogel hybrid	30–50	25–40	3.2–5.0	0.2–0.4	Arid climate	[170]
Conventional (RO desalination)	N/A	10–35	1000+	3.0–4.0	Energy intensive	[6,7,8,9]

**Table 5:** **Passive Cooling and Soil Amendment Performance.** Decomposed performance metrics for passive cooling (evaporative vs. radiative mechanisms) and soil amendments across different soil types. Cooling data shows midday temperature reduction and net cooling power. Soil data quantifies water use efficiency (yield-per-water), nutrient retention (leaching reduction), and material longevity (cycle durability).

Application	Primary Metric	Secondary Metric	Durability	Conditions	References
Passive Cooling-Evaporative	ΔT_noon_ = 8–15 °C (temperature reduction at noon)	Cooling power: 50–150 W/m^2^	2–8 h continuous (40% RH)	20–40% RH, 20–40 °C	[30,31,32]
Passive Cooling-Radiative	ΔT_noon_ = 3–7 °C (temperature reduction at noon)	Cooling power: 40–93 W/m^2^	24 h continuous (no water needed)	Any RH, Clear sky	[217]
Soil Amendment-Sandy	Yield per water: 11–51% increase	Leaching reduction: 30–60%	100–500 cycles (2–3 seasons)	0.2–0.4 g of hydrogel per 100 g of soil	[36,37,38,209]
Soil Amendment-Loam/Clay	Yield per water: 8–25% increase	Leaching reduction: 20–45%	50–200 cycles (1–2 seasons)	0.1–0.2 g of hydrogel per 100 g of soil	[35]
Conventional Systems	AC: 15–25 °C, Drip irrigation: 85–95% efficiency	AC: 2–3 Kw/ton, Irrigation: high capex	AC: 10–15 years, Irrigation: 20–30 years	Energy intensive	[215,218]

## Data Availability

No new data were created or analyzed in this study. Data sharing is not applicable to this article.

## Abbreviations

The following abbreviations are used in this manuscript:

3D	Three-dimensional
4D	Four-dimensional
AA	Acrylic acid
AC	Air conditioning
AI	Artificial intelligence
AM	Acrylamide
ATRP	Atom transfer radical polymerization
AWH	Atmospheric water harvesting
BNNS	Boron nitride nanosheets
CaCl_2_	Calcium chloride
CH_4_	Methane
CO_2_	Carbon dioxide
-COOH	Carboxyl (group)
CuAAC	Copper-catalyzed azide-alkyne cycloaddition
DMPAP	2,2-Dimethoxy-2-phenylacetophenone
ESR	Equilibrium swelling ratio
GHGs	Greenhouse gases
HEMA	Hydroxyethyl methacrylate
HPC	Hydroxypropyl cellulose
HPMC	Hydroxypropylmethyl cellulose
HVAC	Heating, ventilation, and air conditioning
IPNs	Interpenetrating polymer networks
LCST	Lower critical solution temperature
LiCl	Lithium chloride
MBA	Methylene bisacrylamide
ML	Machine learning
MOFs	Metal–organic frameworks
N_2_O	Nitrous oxide
NBW	Non-bound water
-NH_2_	Amine (group)
-OH	Hydroxyl (group)
PAAm	Polyacrylamide
PAAS-PNIPAAm	Polyacrylic acid sodium salt-Poly(N-isopropylacrylamide)
PAM	Polyacrylamide
PEG	Polyethylene glycol
PEG-DA/PEO	Poly(ethylene glycol) diacrylate/poly(ethylene oxide)
PEI	Polyethylenimine
PNIPAM	Poly(N-isopropyl methacrylamide)
PNIPAm	Poly(N-isopropylacrylamide)
PVA	Poly(vinyl alcohol)
PVA/PPy	Polyvinyl alcohol/Polypyrrole
RAFT	Reversible addition-fragmentation chain transfer
RH	Relative humidity
RO	Reverse osmosis
SAHs	Superabsorbent hydrogels
SAPs	Superabsorbent polymers
SBW	Strongly bound water
SDGs	Sustainable Development Goals
T_lum_	Luminous transmittance
TEGDMA	Triethylene glycol dimethacrylate
TiO_2_	Titanium dioxide
UV	Ultraviolet
VO_2_	Vanadium dioxide
VPT	Volume phase transition
WBW	Weakly bound water
ΔT_solar_	Solar modulation ability
